# Pristine graphene covalent functionalization with aromatic aziridines and their application in the sensing of volatile amines – an *ab initio* investigation†

**DOI:** 10.1039/d0ra09964c

**Published:** 2021-02-10

**Authors:** Sabrine Baachaoui, Sarah Aldulaijan, Fayçal Raouafi, Rafaa Besbes, Luca Sementa, Alessandro Fortunelli, Noureddine Raouafi, Adnene Dhouib

**Affiliations:** Laboratoire de Chimie Analytique et Electrochimie (LR99ES15), Departement de Chimie, Faculté des Sciences de Tunis, Université de Tunis El Manar Tunis El Manar 2092 Tunisia noureddine.raouafi@fst.utm.tn; Department of Chemistry, College of Science, Imam Abdulrahman Bin Faisal University Dammam 31113 Saudi Arabia; Institut Préparatoire aux Etudes Scientifiques et Techniques (IPEST), Université de Carthage La Marsa Tunisia; Consiglio Nazionale delle Ricerche, CNR-ICCOM & IPCF Pisa 56124 Italy

## Abstract

Food quality is of paramount importance for public health safety. For instance, fish freshness can be assessed by sensing the volatile short chain alkylamines produced by spoiled fish. Functionalized graphene is a good candidate for the design of gas sensors for such compounds and therefore of interest as the basic material in food quality sensor devices. To shed theoretical insight in this direction, in the present work we investigate *via* first-principles density functional theory (DFT) simulations: (i) graphene functionalization *via* aziridine appendages and (ii) the adsorption of short chain alkylamines (methylamine MA, dimethylamine DMA, and trimethylamine TMA) on the chemically functionalized graphene sheets. Optimal geometries, adsorption energies, and projected density of states (PDOS) are computed using a DFT method. We show that nitrene reactive intermediates, formed by thermal or photo splitting of arylazides – *p*-carboxyphenyl azide (1a), *p*-carboxyperfluorophenyl azide (1b), and *p*-nitrophenyl azide (1c) – react with graphene to yield functionalized derivatives, with reaction energies >−1.0 eV and barriers of the order of 2.0 eV, and open a ∼0.3 to 0.5 eV band gap which is in principle apt for applications in sensing and electronic devices. The interaction between the amines and functionalized graphene, as demonstrated from the calculations of charge density differences showing regions of charge gain and others of charge depletion between the involved groups, occurs through hydrogen bonding with interaction energies ranging from −0.04 eV to −0.76 eV, and induce charge differences in the system, which in the case of *p*-carboxyperfluorophenyl azide (1b) are sizeable enough to be experimentally observable in sensing.

## Abstract 2

Simulations show that the total volatile basic amines (TVB) from spoiled fish can be sensed by aziridine-functionalized graphene nanomaterials to induce substantial changes in the physical properties.

## Graphical abstract

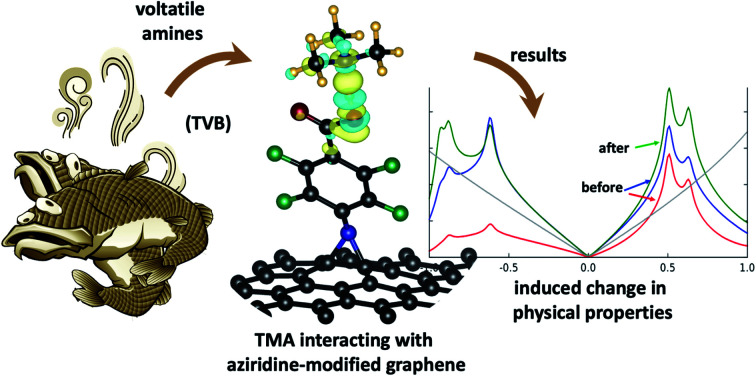

## Introduction

Pristine graphene, a one atom thick layer of carbon arranged in a honeycomb shape 2D crystal, is extensively investigated from theoretical and experimental point of views to unveil its potential applications.^1–6^ Its zero-energy gap however prevents it from being used as such in electronics.^7^ One of the various methods for achieving band-gap opening is chemical functionalization using reactive species such as organic cations, radicals, nitrenes and carbenes.^8–16^ In this respect, reaction with aryl nitrene, generated by thermal or photochemical decomposition of organic precursors,^17^ leads to a [2 + 1]-cycloaddition reaction involving two adjacent carbon atoms from the graphene surface and reactive nitrogen to form an aziridine appended to the graphene.^18–23^ This functionalization causes the re-hybridization of the involved carbon atoms from the sp^2^ to sp^2+*η*^ state (*η*: degree of additional hybridization). Indeed, computational and theoretical studies showed that the pristine graphene functionalization with *p*-azidoperfluorophenyl carboxylic acid leads to a conductivity decrease by *ca*. 50%.^19,24^

Graphene modified with transition elements (Fe, Ni, Co and Cu)^25^ and nitrogen-doped graphene decorated with noble metals such as, Rh, Pt, Pd, *etc.* have been used to develop gas sensors to detect volatile gases such as CH_4_, H_2_S, N_2_, CO_2_.^26^ The organic-functionalized graphene can be obtained *via* chemical and physical adsorption of organic compounds on the graphene surface and be used for gas sensing. For instance, Tang *et al.* used 2,3,5,6-tetrafluorohydroquinone to modify pristine graphene to successfully detect formaldehyde with a good discrimination to ethanol and acetone.^27^ Interestingly, Xu *et al.* designed wearable sensors based on porphyrin-modified graphene and exploited it to detect eight volatile organic compounds (VOCs) using an array of electrodes. The chemoresistance of the devices varies up to 60% in presence of 100 ppm of the VOC vapours.^28^ Non-covalently modified pristine graphene with *N*-substituted triphenylene was successfully used to detect dimethyl methylphosphonate (DMMP), a simulant of the sarin nerve gas, through hydrogen bonding involving the target and the sensing surface. The addition of as low as 1.3 ppm of DMPP induces a variation of the chemoresistance by 10%.^6^

Fish freshness can be assessed using a series of biomarkers^29–33^ such as xanthine employing biosensors,^30–39^ but also by detecting total volatile basic amines (TVB) such as ammonia, methylamine, dimethylamine and trimethylamine, that result from bacterial spoilage, enzymatic lysis and deamination of seafood.^40^ Developing graphene-based sensors with chemical functionalities represents a promising path in this direction since functionalized graphene can lead to a favourable interaction with specific VOCs thanks to the matching chemical characteristics that may result in highly sensitive and specific sensors.

Aiming at providing insight in this direction, in this work, we explore this route *via* theoretical and computational simulations, investigating: (1) the chemical functionalization of graphene nanosheets with aryl nitrenes, obtainable from the decomposition of aryl azides, to yield chemically modified graphene, and (2) the interaction of such chemically modified graphene with typical TVB compounds. In particular, we focused on three modifiers: *p*-azidophenyl carboxylic acid (1a), *p*-azidoperfluorophenyl carboxylic acid (1b), and *p*-nitrophenyl azide (1c), and we found that the functionalization reaction energy is sizeable (>−1.0 eV) for these compounds depending on the added functional groups. Moreover, the formation energy barrier of aziridine-functionalized graphene, estimated *via* a NEB approach, shows transition states at 2.01 to 2.15 eV. The functionalized graphene interacts with MA, DMA and TMA species through hydrogen bonding with energies ranging from −0.04 eV to −0.76 eV (*i.e.* −3.7 kJ mol^−1^ to −73.3 kJ mol^−1^), and the interaction-induced charge differences are appreciable, and in particular in the case of (1b) are of the order of ±0.03/0.06 atomic charge units, correspond to an induced sizeable dipole of few tenths of a Debye, thus being sufficiently large to give rise to an experimentally observable effect.

The article is organized as follows. In Section 2,† we describe the computational approach and the software used to carry out this work. In Section 3,† we present the main results and discuss them in comparison to the relevant literature. In Section 4,† we conclude by emphasizing the main results and the potential application of this work.

### Modelling and computational details

Quantum ESPRESSO (v6.4.1 or v6.5) package^41^ was used to carry out the *ab initio* calculations. For all the DFT calculations, a semi empirical Grimme's DFT-D3 Van der Waals correction was applied.^42^ Projector augmented wave (PAW) basis sets and periodic boundary conditions were employed in the calculations. A generalized gradient approximation (GGA) with the Perdew–Burke–Ernzerhof (PBE) exchange-correlation functional was used. The plane-wave energy cutoffs (ecutwfc) for the wave functions and the charge density (ecutrho) were respectively set at 49.14 Ry and 442.32 Ry (9 × ecutwfc). The latter was optimised for different values of the wave function energy cutoffs (ecutrho = *n* × ecutwfc) where the difference (4 − *n*) × ecutwfc stabilizes (see ESI†).

Each simulated system consists of a supercell corresponding to a 5 × 5 or 6 × 6 graphene unit cells with the three different azide modifiers physically or chemically adsorbed onto it, in the latter case including or not the amine TVB compounds. In the structure relaxation, the Brillouin zone is sampled using a 3 × 3 × 1 Monkhorst–Pack *k*-point grid and the orbital energies are broadened using a Methfessel–Paxton smearing of 0.01 eV, having tested that these parameters give converged results for band structure and electronic properties. For calculating the projected density of states (PDOS), we use a doubled 6 × 6 × 1 Monkhorst–Pack *k*-point grid and a tetrahedron integration method with homogeneous weights on tetrahedra. Atomic coordinates are optimized until the maximum force on ions is less than 0.001 eV Å^−1^ for all systems.

Minimum Energy Paths (MEP) for the reaction between aryl nitrenes and graphene supercell were determined using the Nudged Elastic Band (NEB) method,^43,44^ implemented in the simulation package, connecting the initial and the final equilibrium structures using 21 intermediate images connected with springs. Each image is optimized till the maximum force on ions is less than 0.01 eV Å^−1^ for all systems.

The charge density differences were calculated from the individual charge densities for fully relaxed, minimal energy systems of the adsorbates, substrates and adsorbate/substrate complexes using eqn (1):

1 Δ*ρ* = *ρ*_(substrate+adsorbate)_ − *ρ*_substrate_ − *ρ*_adsorbate_

All atomic and charge density visualisations were made using VESTA software.^45^

## Results and discussion

### Thermodynamics of adsorption and reaction of azides and nitrenes with graphene

#### Adsorption of azides and nitrenes

Starting from fully relaxed, minimal energy configurations of the adsorbates, the 5 × 5 graphene supercell and the adsorbate/graphene, the adsorption energy of phenylazides and phenylnitrenes, *E*_ads_, was computed as the difference between the energy of system (*E*_adsorbate+graphene_) and the energies of the adsorbate (*E*_adsorbate_) and graphene (*E*_graphene_) fragments according to eqn (2):

2 *E*_ads_ = *E*_(adsorbate+graphene)_ − *E*_graphene_ − *E*_adsorbate_

**Fig. 1:**
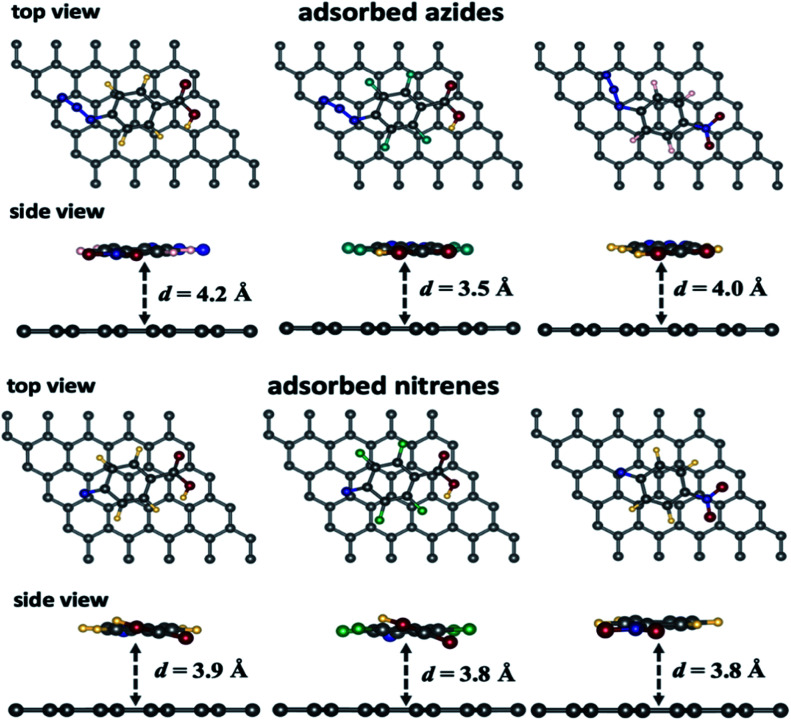
Selected views of the optimized geometries for the different azide/graphene and the corresponding nitrene/graphene from side and top views (colour code for the atoms: H: yellow, C: grey; N: blue, O: red; F: green).

A selection of graphene/adsorbate optimized geometries are displayed in Fig. 1. The physical adsorption and chemical reaction energies of the intermediates with the 5 × 5 graphene supercell are reported in Table 1.

**Table 1:** Adsorption energies and distances separating the adsorbates from the graphene surface^a^

Adsorbates	*E*_ads_/eV	*d*_ads-graphene_/Å
1a	−0.53^b^	4.08^g^
	−0.50^c^	3.45^h^
1b	−0.85^b^	3.97^g^
	−0.90^c^	3.35^h^
1c	−0.29^b^	4.06^g^
	−0.22^c^	3.45^h^
2a	−0.30^d^	3.11
	−0.30^e^	3.19
	−0.31^f^	3.09
	−0.56^b^	3.92^i^
2b	−0.46^d^	3.82
	−0.53^e^	3.08
	−0.55^f^	3.05
	−0.58^b^	3.85
2c	−0.30^d^	3.14
	−0.30^e^	3.20
	−0.31^f^	3.11
	−0.56^b^	3.82
N_2_	−0.20^d^	3.97
	−0.20^e^	3.97
	−0.15^f^	3.97
	−0.21^b^	3.95
g55/3a	−1.04	1.48
g55/3b	−1.10	1.48
g55/3c	−1.09	1.49

a 1a: 4-azidophenyl carboxylic acid; 1b: 4-azidoperflurophenyl carboxylic acid; 1c: 4-nitrophenylazide; 2a: 4-carboxylphenylnitrene; 2b: 4-carboxylperflurophenylnitrene; 2c: 4-nitrophenylazide; g55/3a: 4-carboxylphenylaziridine appended to a 5 × 5 graphene supercell; g55/3b: 4-carboxylperflurophenylaziridine; g55/3c: 4-nitrophenylaziridine.

b Parallel position.

c Vertical position.

d Atop position.

e Bridge position.

f Hollow position.

g Mean distance to graphene surface.

h Closest atom to graphene.

i Ref. 48.

**Table 2:** Selected geometrical features for the aziridine appendages tethered to the graphene surface

Substrate	*h*/Å	*d*_1_/Å	*d*_2_/Å	*α*/°	*β*/°	Tilt angle
3a	0.62	1.55	1.48	117.6	58.4	53.8
3b	0.63	1.55	1.48	117.5	58.4	52.1
3c	0.61	1.55	1.47	117.6	58.4	53.7

From Table 1, we can observe that the arylazide derivatives are located at a distance ranging from 4.0–4.1 Å and 3.4–3.5 Å parallel and vertical to the surface, respectively. The closest one is the *p*-azidoperfluorophenyl carboxylic acid (1b), which the most electron deficient species due to electron withdrawing by the fluorine atoms. Moreover, we can observe that all the nitrene intermediates lie at a shorter distance from the graphene surface *ca*. 3.1–3.2 Å when they are in atop, bridge and hollow positions. In the parallel position, the intermediates are lying at a slightly larger distance from the surface (*ca.* 3.8–3.9 Å) because of larger repulsion between the electronic clouds. Energetically, the parallel position yields the strongest adsorption energies comparatively to the other configurations because a larger number of atoms from the absorbates are facing the graphene. The 2b nitrene gives the highest adsorption energies because of the fluorines on the aromatic ring. Finally, the parallel configuration seems to favour adsorption compared to the vertical configuration (entries 2 and 6, Table 1) while the perfluorinated compound is the least adsorbed on the surface compared to the two others. Furthermore, in case of N_2_, according to the energy data, the most favoured position is the one parallel to graphene, while the hollow position lies at 0.06 eV higher than the parallel one.

#### Reaction of nitrenes with graphene

The nitrene intermediates react with a carbon double bond from the graphene to yield 4-substituted aziridine/graphene adducts.^18,46^ The calculated functionalization reaction energy values are close in the three cases and are in the range between −1.04 and −1.10 eV (100.3 to 105.8 kJ mol^−1^), which is consistent with reaction energies reported in literature.^47^ These high values are indicative of a chemical reaction occurring between graphene C=C and the nitrogen from the nitrene intermediates to give three atom rings *via* a [2 + 1] cycloaddition reaction. The most electron deficient nitrene intermediates 2b and 2c seem to react more favourably with the graphene surface than 2a to form g55/3a–c since the *E*_ads_ for the latter is 60 meV lower than the two others. For instance, 2a is more stable than 2b since the latter bears four electron withdrawing fluorine atoms and is formed at slightly higher energy than 2a (*cf.* NEB plots in Fig. 4).

**Fig. 2:**
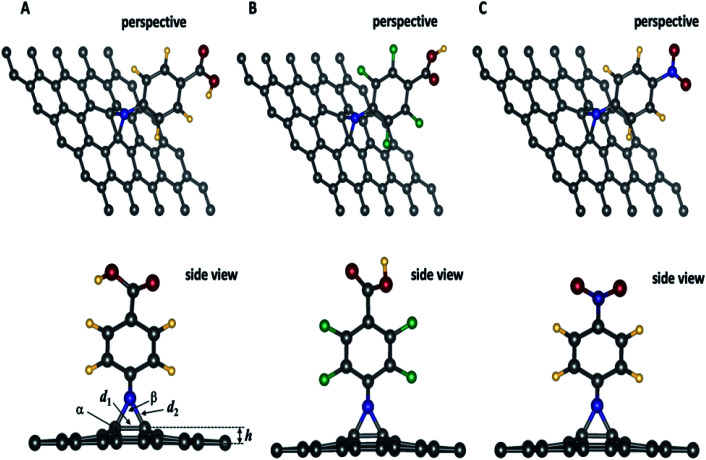
Optimized geometries of 4-substituted aziridine–graphene nanosheets. In the side view, the arrow indicating different geometrical features summarized in Table 2. Legend: elevation: h; *d*_1_: distance: C–C; *d*_2_: distance C–N, *α*: angle CCC; *β*: CNC.

**Fig. 3:**
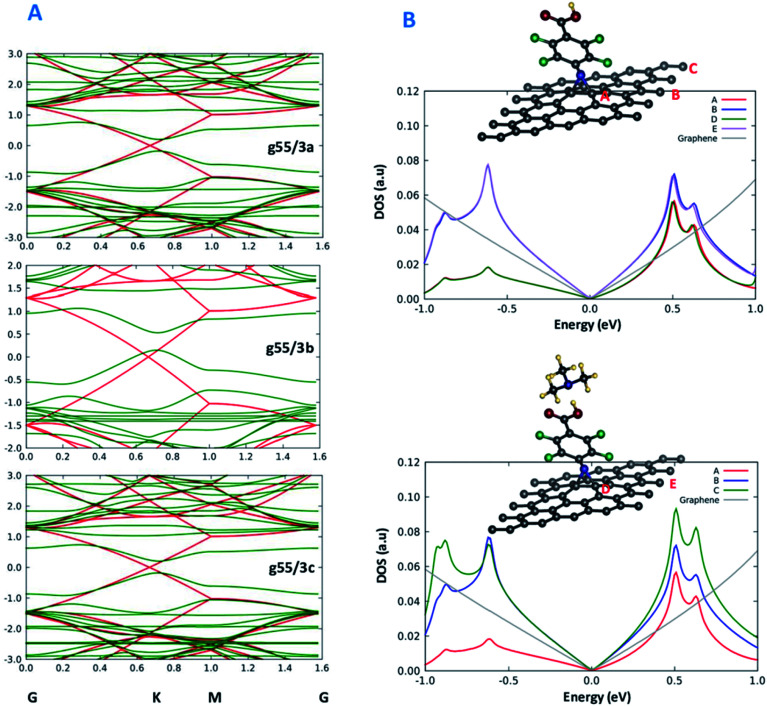
(A) Band structure plots of the modified graphene g55/3a, g55/3b and g55/3c (green) comparatively to unmodified graphene (red) and (B) PDOS plots of the interaction between the functionalized graphene and amine species to be sensed. Curves A and B of the bottom panel are also reported in the top-panel for the sake of comparison.

**Fig. 4:**
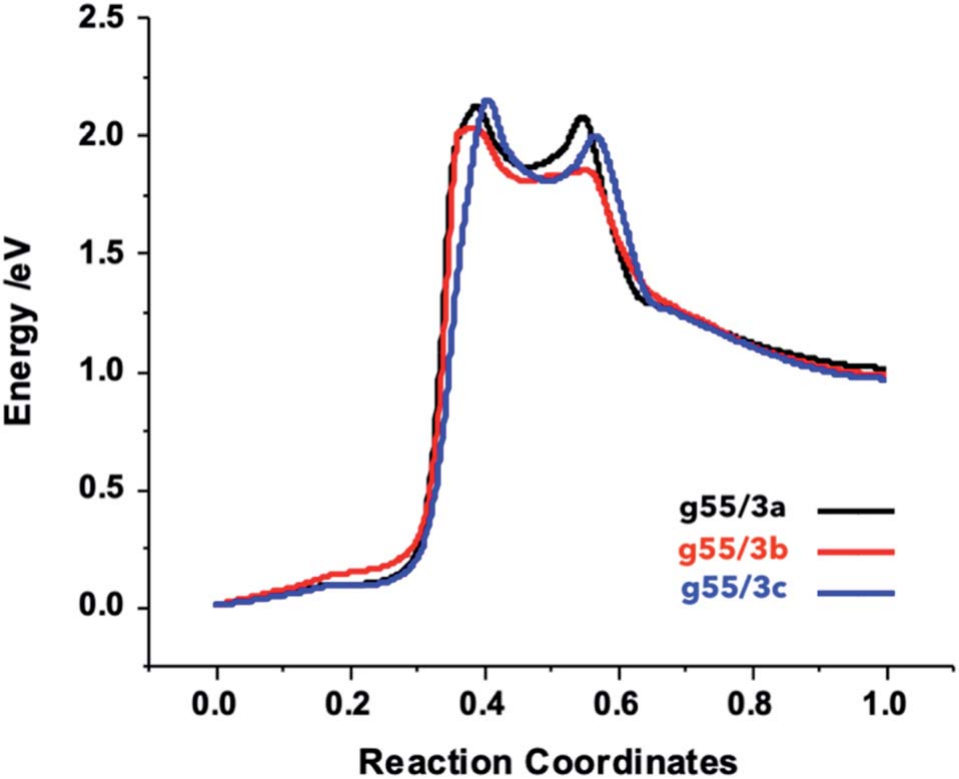
NEB plots of the reaction between the functionalized arylazides and the graphene.

### Geometrical features of 4-substituted aziridine-graphene nanosheets

Fig. 2 presents the optimized geometries for the three aziridine-tethered graphene substrates. We can immediately observe a protrusion of two graphene carbon atoms out of the graphene plane following the formation of the aziridine ring (side view). This is due to the change in hybridization of these atoms from sp^2^ to sp^2+*η*^ state, where *η* is the degree of additional hybridization. The aziridine-induced deformation propagates to the surrounding carbon atoms and fades away along the axes. The reactive N from nitrene undergoes a cycloaddition reaction to yield the corresponding aziridine cycle. The values of C–N and C–C bonds in the aziridines rings are respectively 1.47 to 1.48 Å and 1.55 Å, and are consistent with the usual values for sp^3^ hybridized N and C atoms and with those from literature using DFT and the B3LYP and B3LYP/ωB97XD^47^ exchange-correlation functionals. Moreover, Table 2, reporting the geometric features of functionalized graphene, shows that the formation of an aziridine ring involving carbon atoms from the graphene induces a protrusion of these carbons from the surface of the graphene sheet by approximately ≈0.6 Å, thus confirming the partial change in carbon hybridization, which is also confirmed by the value of the angle linking the involved carbon to two adjacent ones (117.6°). The aziridine ring is constrained since the CNC angle is 58.4° that is close to 60° as in small-constrained cycles.

### Band structure and projected density of states

To achieve a deeper understanding of the changes in transport properties of graphene brought about by chemical functionalization, we analyse in detail the band structure of the systems. Fig. 3A shows the band structure of the three functionalized graphene systems along a path in *k*-space going through the Dirac point of graphene. Interestingly, it is apparent from Fig. 3A that a band gap opens in all three cases along the path, approximately ranging in size from 0.3 eV to 0.5 eV. This result is in nice keeping with previous theoretical and experimental work of Plachinda *et al.*,^19^ and suggests that these systems can be suitable for sensing applications due to the electron mobility degradation along specific directions of the Brillouin zone. We add that this effect will certainly depend on the density of the modifiers on the surface. This can be better appreciated from an analysis of the density of states (DOS) projected onto various carbon atoms (PDOS) illustrated in Fig. 3B, where a difference can be appreciated between the DOS projected onto carbon atoms closer or farther from the modifier, which are therefore more or less affected by the structural deformation induced by functionalization. Indeed, the experimental results reported in Fig. 8 of ref. 19 show that the reduction in conductance around the Fermi energy (zero bias) is less pronounced than that predicted by theory, although it is still present at a level sufficient for applications, which we interpret as due to a too high density of modifier in the theoretical models with respect to experiment. Fig. 3B thus suggests that the density of modifiers is a parameter to be taken into account in experiment to precisely modulate the sensing capability of these systems.

### Functionalization mechanism

In order to gain more insights into the path leading to the formation of aziridine–graphene adducts, we determined the NEB profiles for the different reactions (Fig. 4). The plots show a first energy barrier located at coordinates *ca.* 40% of the reaction path with an energy of *ca.* 2.1 eV corresponding to the splitting of the azylazide into arylnitrene and nitrogen, which in fair agreement with the experimental value of the formation of arylnitrene from arylazide determined in liquid phase (163 kJ mol^−1^, 1.7 eV)^49^ – liquid phase effects in experiment will likely reduce the gas-phase energy barrier we determine here. A second transition state corresponding to the approach of arylnitrene to graphene is located at 55% of the reaction path and an energy of 2.0 eV. Finally, a descendent path leads to the final products, which lie at a higher energy with respect to the starting reagents. This confirms that an initial energy activation (by photolysis or thermolysis) is necessary to overcome the energetic barrier of the arylnitrene formation,^50^ which reacts rapidly with the carbon–carbon double bond from graphene to yield the final products. In experiment, the products are stabilized by the evolution of nitrogen gas and the associated free-energy gain (more than 1 eV at 400 °C), which overcomes the electronic energy cost of the functionalization reaction. It is worth mentioning that in case of the g55/3b adduct the barrier is lower by 14.5 kJ mol^−1^ (0.15 eV) comparatively to the two others due to electronic effect induced by the fluorine atoms.

### Gas sensing

#### Thermodynamic data

**Fig. 5:**
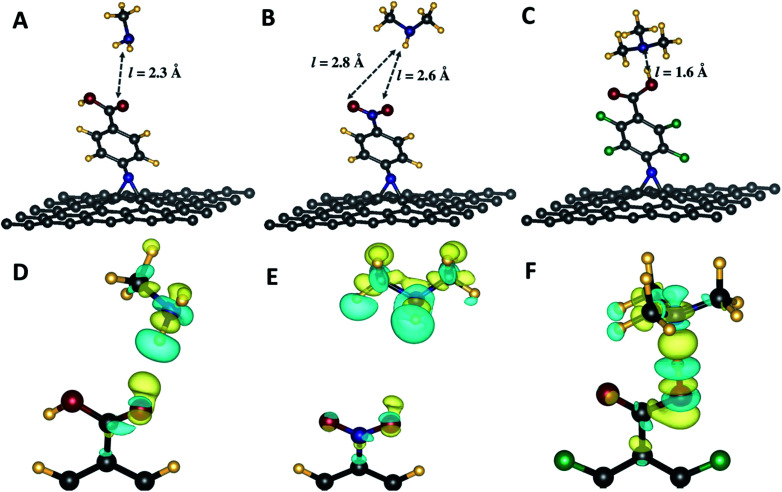
(A–C) Optimized geometries different amines interacting with aziridine-appended graphene sheets. Arrows indicating the lengths of hydrogen bonding established between the amines and the modified graphene. (D–F) Plots of charge density differences showing regions of charge gain (yellow) and regions of charge depletion (blue) involving the functional groups on the graphene surface and the sensed amines. Values of the isosurface plots for the charge density differences are (D): 7.5 × 10^−4^, (E): 1.5 × 10^−4^ and (F) 2.5 × 10^−3^ e Bohr^−3^.

The sensing properties of the aziridine-appended graphene substrates as materials to construct sensing devices have been investigated using DFT augmented by D3-Grimme for long range dispersion corrections.^42^ The relaxed structures, pictorially illustrated in Fig. 5, show that the functional groups (–CO_2_H, –NO_2_) interact with N–H or C–H groups from the amines through hydrogen bonding. The lengths of such hydrogen bonds are comprised between 1.6 Å and 2.8 Å respectively, being O–H⋯N in the case of *N*-tetrafluoroaphenyl aziridine and N–H⋯O=N in the case of *N*-nitrophenyl aziridine. Furthermore, as shown in Table 3, the interaction energies vary in a wide range, from −0.04 and −0.76 eV respectively for CH⋯O and N–H⋯O bonds between the C–H from the tertiary amine and the N–H and C=O from the carboxylic acid, respectively. Finally, it is noteworthy that the tertiary amine interacts more strongly with the carboxylic acid group from the phenyl carboxylic acid and perfluorophenyl carboxylic acid. The weakest hydrogen bond was noticed with g55/3c substrate which does not present and acidic group.

**Table 3:** Interaction energies, atoms involved in the hydrogen bonds and their lengths

Adsorbates	Substrates	Energy/eV	HB lengths/Å
MA	g55/3a	−0.57	NH⋯O: 2.29
	g55/3b	−0.09	OH⋯N: 1.60
			NH⋯O: 2.48
	g55/3c	−0.16	NH⋯O: 2.59
			NH⋯O: 2.88
DMA	g55/3a	−0.16	NH⋯O: 2.21
	g55/3b	−0.67	OH⋯N: 1.55
			NH⋯O: 2.84
	g55/3c	−0.14	NH⋯O: 2.65
			NH⋯O: 2.73
TMA	g55/3a	−0.75	OH⋯N: 1.64
	g55/3b	−0.76	OH⋯N: 1.57
			CH⋯O: 2.71
	g55/3c	−0.04	CH⋯O: 2.75

#### Lowdin charges

Next, we focus on a Lowdin charge analysis following the adsorption process to extract information on the sensing capability of the proposed systems. We analysed the charge density differences between the system before and after adsorption of the tertiary amine as a prototypical case, and found that for g55 substrate only minor differences of −0.002*e* on the H closest to the O of the CO_2_H group can be observed.^51^ Somewhat larger differences were found for g55/3c where N and the two H closest to the NO_2_ lost −0.006 and −0.004*e*, respectively, in favour of the 2H (+0.004*e*) farthest from the aromatic functionalizer. Finally, for g55/3b, we found that the CO_2_H group gets a large additional electron charge (+0.04*e* for O of the OH, +0.03*e* for the other O, and +0.06 for H), yielded −0.05*e* from N and −0.06 from a H of the methyl groups far from the aromatic functionalizer, corresponding to an induced significant dipole of few tenths of a Debye and therefore to a local drop in electrostatic potential of several tens of mV, sufficiently large to give rise to an experimentally observable effect. Although also the g55/3c pair can be of interest, the g55/3b pair thus seems the most favourable case for sensing as it exhibits the largest adsorption-induced dipole, both in terms of absolute values of the charge differences and in terms of distances between the so-generated charges.

#### Charge density differences

Furthermore, to evidence the interactions between the different amines and the aziridine-modified graphene derivatives, we start from fully relaxed, minimum-total-energy configurations and we compute the charge density difference for the three cases depicted in Fig. 5A–C, which were chosen because they show the highest HB energies (entries 1, 5 and 7 of Table 3). The areas showing the charge density variations are represented in Fig. 5D–F. We can see regions of charge gain (yellow colour) around the oxygen atoms from CO_2_H and NO_2_ groups involved in the hydrogen bonding concomitantly with regions of charge depletion (blue colour) on the hydrogen from the NH groups of MA and DMA. In case of the interaction between g55/3c and TMA, the charge depletion occurs on the acidic hydrogen from the graphene substrate, while the charge gain is observed on the nitrogen from the TMA adsorbate. The effect is less pronounced in case of g55/3a with MA and g55/3b where the isovalues of the charge density difference read several times lower than the case of g55/3b with TMA (highest HB energy). These results are in good agreement with the computed thermodynamic data and Lowdin charges.

## Conclusions

Graphene functionalization is of great interest in current physico-chemical research in view of sensing applications, with many potential applications such as *e.g.* in the detection of volatile (short-chain) amines for food safety. In the present work, we show that pristine graphene can be modified with various nitrene intermediates, generated from the decomposition of azides, to yield the corresponding aziridine-modified graphenes, representing a novel path to functionalized graphene materials. Such reactions are thermodynamically favoured and occur with a reaction energy ranging from −1.04 to −1.10 eV. A combined band-structure and PDOS analysis show the opening of an energy band gap yielding a semi-conducting state of graphene, which make it suitable for transistor applications, however pointing to a dependence of this phenomenon upon the density of modifiers as a key parameter. Our modelling finally suggests that some of the resulting materials (specifically, those generated by the perfluorophenyl azide modifier) can be used to sense short-chain aliphatic amines: their interaction *via* hydrogen bonding with the functional group anchored onto the graphene surface induce appreciable charge differences upon adsorption which should be experimentally detectable, *i.e.*, generate electrostatic perturbations strong enough to be a good candidate for sensing applications. The concept here explored can be extended to a whole class of host–receptor pairs adsorbed or chemically linked to the graphene surface, thus representing an appealing path to exploit the unique properties of 2D graphene-based materials.

## Conflicts of interest

There are no conflicts to declare.

## Supplementary Material

RA-011-D0RA09964C-s001
