# Supplementary material for: Pristine graphene covalent functionalization with aromatic aziridines and their application in the sensing of volatile amines – an ab initio investigation
Source: RSC Adv. 2021 Feb 10;11(12):7070–7. doi: 10.1039/d0ra09964c (PMC8694903; doi:10.1039/d0ra09964c)
Supplement: RA-011-D0RA09964C-s001 [file RA-011-D0RA09964C-s001.pdf]

Electronic Supplementary Material (ESI) for RSC Advances.
This journal is © The Royal Society of Chemistry 2021

Electronic Supplementary Information

Pristine graphene covalent functionalization with aromatic aziridines and their application in the sensing of volatile amines – An ab initio investigation

Sabrine Baachaoui,[a,b] Sarah Aldulaijan,[b] Fayçal Raouafi,[c] Rafaa Besbes,[a] Luca Sementa,[d] Alessandro Fortunelli,[d*] Noureddine Raouafi[a*] and Adnene Dhouib[b*]

Optimization of the charge density energy cutoff

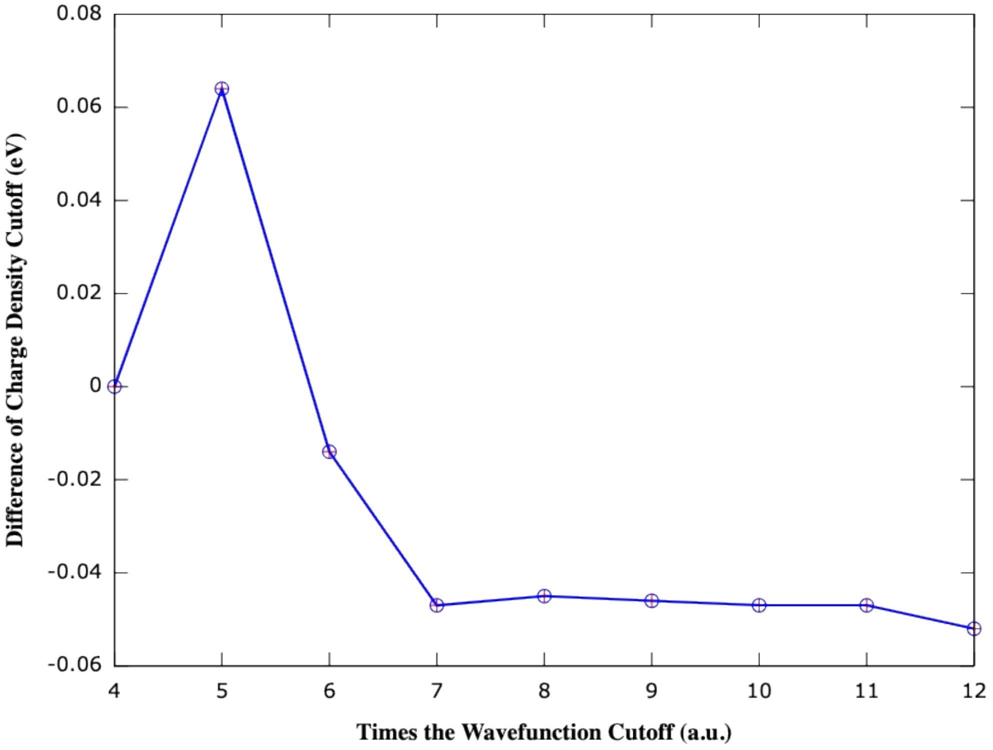

Figure S1. Variation of the difference of the charge density energy cutoff (EcutRho) in function of the number of the wavefunction cutoff (EcutWFC) showing stabilization of the difference above EcutRho = 7×EcutWFC.

Optimization of the supercell size

Table S1. Adsorption energies for the 5×5, 6×6 and 7×7 graphene supercells in presence of 2b adsorbate and per elementary cell of graphene

	5×5 Supercell	6×6 Supercell	7×7 Supercell
E_{ads}/supercell	-0.72	-0.84	-0.97
E_{ads}/elementary cell	-0.029	-0.023	0.020

Charge density difference plots

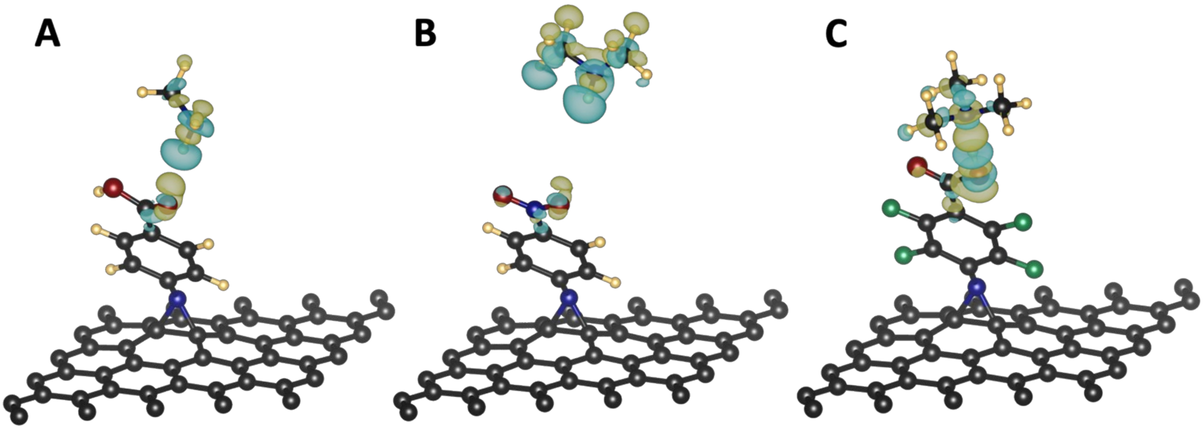

Figure S2. Selected views for the charge density differences brought about by the interaction of functionalized graphene with an amine computed for A) g55/**3a** with methylamine, B) g55/**3b** with dimethylamine and C) g55/**3c** with trimethylamine (yellow: regions of charge gain and blue: regions of charge depletion). Values the isosurfaces for the plots of charge density differences are: (A) 7.5×10^{-4}, (B) 1.5×10^{-4} and (C) 2.5×10^{-3} e/Bohr^3.
